# Obituary

**Published:** 1871-09

**Authors:** 


### OBITUARY.

Died—At the residence of her parents, Riverside, on the Cincinnati & Indianapolis railroad, August 3d, 1871, Sallie, infant daughter of Dr. William and Rebecca Taft, aged 18 months.

Death, at no time, a welcome visitor, seems most unwelcome in the season of infancy, when the tender bud is beautiful and full of promise. The special claims of infancy upon the affections and devotion of the heart, are enhanced by the utter dependency and innocence embodied in the little creature that can only look its simple desires, but when the early blight falls upon it, and all that was invoked for its care and protection, is turned to bitterness and disappointment, the consolations of religion only can heal the wound that death inflicts, because the soul is lifted into companionship with the blest, and glimpses of the Eternal rest and union are granted by the merciful Father, who pitieth all those who love Him.

